# Pavlovian to instrumental transfer of control over fight or flight decisions

**DOI:** 10.1038/s41539-025-00331-4

**Published:** 2025-05-28

**Authors:** Andreas B. Eder, Vanessa Mitschke

**Affiliations:** 1 University of Würzburg (JMU), Institute for Psychology, Würzburg, Germany; 2 University of Göttingen, Institute for Psychology, Göttingen, Germany

**Keywords:** Human behaviour, Human behaviour

## Abstract

This study investigated outcome-selective Pavlovian-to-instrumental transfer (PIT) in fight-or-flight decision making. Participants learned to attack or retreat from monsters (instrumental phase) and to associate environments with specific monsters without responding (Pavlovian phase). In the transfer phase, they chose responses to unseen monsters while exposed to conditioned stimuli (CSs). Study 1 (*n* = 86) found that CSs influenced fight-or-flight decisions, demonstrating both outcome-selective and outcome-general PIT effects. Study 2 (*n* = 76) tested the operation of cognitive beliefs with post-training instructions that reversed the CS-outcome relations, revealing a reversed PIT effect. Study 3 (*n* = 83) manipulated threat levels by featuring highly dangerous monsters. Results showed a larger specific PIT under low versus high threat with standard instructions but not with reversal instructions. Findings suggest that associative knowledge about upcoming threats is integrated with knowledge of defensive actions into cognitive beliefs about which response is most effective for coping with danger.

## Introduction

Organisms survive and thrive because they have developed defensive strategies against upcoming challenges and threats that are inferred from cues in the environment. These cues for defensive action can be evolutionarily fixed, such as the smell of a fox to a rat, or they can be rapidly learned through experiences indicating that a specific place, time, or object is dangerous^1,2^. The latter form of learning is extensively studied through aversive conditioning procedures, in which, by repeated pairings, a neutral stimulus (the conditioned stimulus, CS) becomes predictive of an aversive outcome (the unconditioned stimulus, UCS). Conditioned (‘Pavlovian’) signals for specific threats were found to affect the preparation for active defensive (‘operant’) behavior—a phenomenon that has been intensely studied using the Pavlovian-to-instrumental transfer (PIT) paradigm^3^.

The PIT paradigm is designed to investigate how a CS associated with a specific outcome can influence an instrumental response linked to the same outcome. Most PIT tasks utilize rewards such as food, drugs, or money as outcomes^4^. A typical PIT task consists of three separate phases: (1) The *Pavlovian Conditioning Phase* is used to establish associations between specific stimuli (the CS) and specific outcomes (the US) by pairing them. For example, a tone (CS1) is paired with pellets of Food A (Outcome 1), a light (CS2) is paired with pellets of Food B (Outcome 2), and a clicker (CS3) is paired with pellets of Food C (Outcome 3). (2) The *Instrumental Conditioning Phase* is used to establish associations between different instrumental responses and specific reinforcers. For example, pressing Lever 1 (R1) is reinforced with pellets of Food A (Outcome 1) and pressing Lever 2 (R2) is reinforced with pellets of Food B (Outcome 2). (3) In the *Transfer Test Phase*, both actions (R1 and R2) are made available in Pavlovian and instrumental extinction (i.e., without presentation of outcomes). Response frequencies are then measured during the presentations of the Pavlovian cues. A typical finding is that a response is produced more often, or more vigorously, in the presence of a cue that was predictive of the same outcome (CS1:R1, CS2:R2) compared to stimuli associated with a different outcome (CS1:R2, CS2:R1) or baseline periods with no stimulus presentations^5,6^. This finding is labeled “outcome-specific PIT effect” because it is driven by expectancies of specific rewards (here: foods). Another common finding is that both reinforced responses are produced more often during presentations of the CS3, paired with a reward not used as an operant reinforcer, relative to a baseline period or neutral comparison stimuli (CS-)^7,8^. This finding is dubbed the “outcome-general PIT effect” because it is explained by the activation of an outcome belonging to the same motivational (here, the ‘appetitive’) class.

More recently, researchers have also begun to develop aversive PIT task variants using the avoidance of intrinsic (e.g., pain) and symbolic threats (e.g., financial loss, monsters) for reinforcement. As reviewed by Campese^3^, the development of aversive task variants was neglected for a long time due to a theory-driven shift to purely Pavlovian studies in the eighties and significant challenges in studying avoidance learning. Specifically, successful avoidance typically involves the omission of a threatening outcome, such as pain, meaning the outcome of avoidance is characterized by the absence of the sensory event constituting the threat (e.g., no pain). Therefore, avoidance learning usually involves a warning signal to create a negative prediction error, where performing the instrumental response prevents the US predicted by the warning signal. However, using warning signals of specific threats during the instrumental training phase can complicate the implementation of PIT tasks, as they may act as discriminative cues for selecting a specific avoidance response. These challenges, at least in studies with humans, can be addressed by using symbolic threats that can be visualized on the screen (e.g., attacking monsters) and by using a conditioning procedure known as the Sidman Avoidance Schedule^9^. In this procedure, the subject experiences periodically an aversive event (e.g., pain every 5-sec) unless a specific response is performed. If the response is made, the aversive event is postponed by a fixed amount of time (e.g., by an additional 30-sec) from that response. The avoidance response is then reinforced by reducing the overall rate of the aversive event without explicit presentations of CSs warning of the US. Instead, the time elapsed since the last US effectively functions as a warning signal.

Importantly, this procedure can be integrated in aversive PIT tasks for learning to avoid distinct threats. A specific example is provided by the study of Lewis and colleagues^10^ (for similar procedures see refs. ^8,11^). Using the Sidman reinforcement procedure, human subjects first learned to fend off attacks of specific monsters (e.g., goblin, ogre or troll attacks; O1-O3) with particular keypresses (R1-R3). In the subsequent Pavlovian conditioning phase, they learned to expect attacks of specific monsters, including a novel monster not shown in the previous phase, that were contingent upon presentations of specific visual cues (CS1-CS5). Subsequently, participants pressed keys of their choice during presentations of the visual cues in extinction (i.e., without attacking monsters). Results showed a specific PIT effect: instrumental responding to the conditioned stimuli was selectively increased when the CS signaled a monster attack that was used to negatively reinforce the defensive action. Results also demonstrated an elevated response rate relative to baseline when the CS signaled a monster attack that was not negatively reinforced in the Sidman phase (i.e., a general PIT effect). Functional neuroimaging additionally showed that both effects correlated with increased activation in corticostriatal circuits, most notably the striatum and the cingulate cortex, that are also active in appetitive PIT task variants^4,12^. Accordingly, related neuropsychological processes are presumably operative in appetitive and aversive PIT tasks with humans^3^.

Empirical studies on PIT effects inspired several theoretical explanations that differ in their emphasis on the nature of involved processes and mental representations. Dominant theories could be classified based on whether they emphasize associative or propositional learning processes. Associative theories claim a network of associations that link CS to outcomes after Pavlovian learning (S-O associations) and responses to outcomes after instrumental learning (R-O associations)^13,14^. Due to the bi-directionality of the R-O association, the CS activates a specific response via priming of the shared outcome. This S-O-R chain explains specific PIT effects with activation of sensory components of outcomes. Moreover, the link to a sensory representation, without access to value codes, can explain why specific PIT effects were often, but not always, found to be insensitive to post-training changes of outcome values^15,16^. The general PIT effect is explained with associative links to two general motivational systems, one regulating appetition (reward, consumption, etc.) and the other one aversion (avoidance, defense, etc.)^17^. Activation of one system primes interconnected responses of the same motivational class and interferes with responses associated with the other motivational class^18^.

Propositional theories, in contrast, propose knowledge structures that specify directional relationships between elements^19^. For example, after repeated pairings of a CS with an aversive US, an expectancy may be formed that the CS causes or signals the US. Avoidance is motivated by the belief that the signaled US could be prevented or is less likely to follow the CS when the avoidance response is emitted^20,21^. This propositional account can explain specific PIT by proposing that (non-)humans can form new propositions via inferential reasoning^15^. For example, when a dog expects a shock sensation to its right hindleg after Pavlovian conditioning, and has learned to terminate a shock to the right leg by tilting his head to the right, he will likely infer for the transfer phase that he must move his head to the right when the warning signal occurs even when this signal was never directly paired with the avoidant action during the learning sessions^22^. The propositional account also claims that propositions can be acquired through verbal instructions. In support of this, Seabrooke and colleagues^23^ trained human research subjects to work for beer and chocolate outcomes. When standard instructions were used, presenting pictures of beer and chocolate products during the transfer phase selectively increased the vigor of working for beer and chocolate, exhibiting a specific PIT effect. However, when participants were informed that the pictures would signal which response would *not* be rewarded, the specific PIT effect was reversed. This reversal suggests that the research participants have inferred the appropriate response based on the given instruction, forming a new propositional belief for the transfer phase (e.g., “To earn beer, I must press the beer response key when I see a chocolate picture. To earn chocolate, I must press the chocolate response key when I see a beer picture.”).

Of course, one can also claim the dual operation of both, associations and cognitive (propositional) beliefs^24^. While tempting, this dual-systems approach often leads to excess theorizing, which requires scientific explanation not only of the two claimed systems but also of their interaction. For these inquiries, theories often propose distinct operating characteristics for associations and propositions, assuming that association formation and their use for action is more automatic and efficient than rule-based behavior^25,26^. This assumption can be tested with manipulations that deplete the necessary cognitive resources for the formation and/or employment of propositional beliefs. For example, Seabrooke and colleagues^27^ manipulated cognitive load in a series of studies in addition to providing (reversal) instructions for the transfer phase. When participants received standard instructions for the test phase, a specific PIT effect was observed even in a condition with high cognitive load. In contrast, the specific PIT was reversed by the reversal instruction only when there was no cognitive load. Hence, high cognitive load eliminated the instruction-induced reversal of the PIT effect, suggesting that the use of this instruction was presumably more complex and therefore less efficient.

Other studies investigated the influence of personality factors related to impulsivity and stress on specific and general transfer. Quail and colleagues^28^ found that participants, who indicated high levels of self-reported chronic stress and anxiety, worked more vigorously for rewards (general PIT effect), whereas the magnitude of the specific PIT effect was (non-significantly) reduced in tendency. Hinojosa-Aguayo and González^29^ found a negative correlation between individual differences in affect-driven impulsivity, as assessed with a self-report measure, and the magnitude of specific PIT effects. They concluded from their studies that outcome-specific transfer is most likely based on a ‘cognitive’ process that is impaired by high levels of chronic stress and impulsivity, whereas general transfer is mostly driven by associations and therefore not impaired.

In the present study, we were specifically interested in the cognitive processes that motivate action decisions in humans to flee or fight (i.e., in the cognitive processes underlying specific PIT effects in the aversive domain). As reviewed above, most PIT studies with humans have investigated specific transfer effects in the appetitive domain, using food and money as reinforcers, and leaving the control of aversive behaviors largely understudied. Accordingly, more research is needed on the cognitive control processes of defensive behaviors that integrate information from environmental cues. We were specifically interested in fight-or-flight decisions because these constitute the fundamental decision to seek or avoid the confrontation with a source of threat. Classic research on the fight-or-flight response studied vegetative responses of the sympathetic nervous system (e.g., increases in blood flow, increased availability of oxygen and glucose) as an “alarm reaction” that mobilizes the organism for defensive action when exposed to a conspecific or predator^30^. Hence, this research had an almost exclusive focus on passive (Pavlovian) reactions. In the present study, we used an aversive PIT task to study the cognitive processes that integrate information from aversive cues signaling threatening events in action decisions to flee or fight.

For obvious ethical reasons, we did not expose our research participants to real threats, only to symbolic threats (digital monsters). The PIT task was modeled after the procedure Lewis and colleagues^10^. Specifically, participants first learned a defense action in a Sidman avoidance procedure by either fleeing from or fighting against a specific monster. In the subsequent Pavlovian phase, they learned to associate specific environments (CS) with the appearance of a specific monster that posed a threat of attack. In the final transfer phase, participants had the choice between fight or flight when exposed to the environments but without seeing the monsters (formally corresponding to an extinction phase that prevents new learning). Table 1 summarizes the experimental contingencies in each phase.

**Table 1:** Summary of experimental contingencies

Instrumental Training	Pavlovian Training	Transfer Test
Key ↑ → fight (Monster A) Key ↓ → flight (Monster B)	CS1→ Monster A	CS1: Key ↑ *vs* Key ↓
	CS2→ Monster B	CS2: Key ↑ *vs* Key ↓
	CS3→ Monster C	CS3: Key ↑ *vs* Key ↓
	CS4→ Monster D	CS4: Key ↑ *vs* Key ↓
	CS5→ Object 1	CS5: Key ↑ *vs* Key ↓
	CS6→ Object 2	CS6: Key ↑ *vs* Key ↓

Instrumental training involved pressing arrow keys to either attack or retreat from a monster. The conditioned stimuli (CS) were six distinct environments, each associated with the appearance of either a specific monster or a specific neutral object. Two CS predicted monsters that were featured in the fight-flight animations during the instrumental training phase (CS + _AB_), two additional CS predicted novel monsters not previously encountered (CS + _CD_), and two CS signaled the appearance of a neutral object rather than a monster (CS-). During the transfer test, no Pavlovian or instrumental outcomes were shown. For further details, refer to the Method section.

We recorded fight-or-flight decisions during the transfer phase as a function of CS presentation, hypothesizing a preference for actions associated with the same defensive outcome compared to a different outcome. Specifically, participants should prefer fighting (over fleeing) when the CS + _A(fight)_ signals a monster that is attackable, and vice versa when the CS + _B(flight)_ signals a monster that can be fled from (i.e., an outcome-specific PIT effect).

Before describing the specific experiments, it is important to emphasize that our aversive PIT task variant is not a simple mirror image of typical reward-based PIT tasks. The two task variants differ not only in the type of elicited motivation (appetitive vs. aversive) but also in the conceptualization of outcomes. In reward-based PIT tasks, outcomes are typically identical across the Pavlovian and instrumental conditioning phases—that is, the same reward is delivered both when signaled by a Pavlovian cue and following an instrumental response. In contrast, our aversive PIT task involves different outcomes in the Pavlovian and instrumental phases. During the Pavlovian phase, participants learn to expect a threatening event (i.e., the appearance of a dangerous monster) based on a cue. In the instrumental phase, however, they learn to expect the *omission* of being hit by a specific monster contingent upon performing a particular response. Thus, while the outcomes are partially overlapping—given the involvement of the same monster across phases—they are not identical.

We do not regard this asymmetry as a conceptual limitation. On the contrary, we propose that interactions between Pavlovian expectancies of aversive events and instrumental expectancies of coping with these events are characteristic of real-world scenarios outside the laboratory. Accordingly, our task should be understood as a model of aversively motivated Pavlovian-instrumental interactions in ecologically plausible settings, rather than as a direct analogue to reward-based PIT tasks.

We report a total of three experiments. Experiment 1 served two primary purposes. First and foremost, it aimed to demonstrate an outcome-specific (“fight-*or*-flight”) PIT effect: when a monster attack is signaled by a CS, participants should more often select the defensive action that was previously learned as an effective defense against this specific monster compared to the alternative response. For this comparison, we considered only those trials in which cues predictive of the monsters featured in the instrumental phase were presented (i.e., trials featuring CS + _AB_).

Second, the procedure was adapted to also test for an outcome-general PIT effect. To this end, we included additional aversive cues associated with novel attacking monsters (i.e., trials featuring CS + _CD_), which were expected to increase overall defensive responding (“fight-*and*-flight”) relative to a baseline condition with neutral cues (the CS-). Thus, Experiment 1 was designed to demonstrate both types of transfer: outcome-general and outcome-specific.

After establishing a paradigm that demonstrates a specific PIT effect, we conducted further experiments to study the underlying processes of cue-motivated fight-or-flight decisions. Seabrooke and colleagues^23^ found that providing instructions for the transfer phase, stating that the CS would signify which response would *not* be rewarded during this phase, reversed the outcome-specific PIT effect. This reversal was interpreted as supporting an explanation involving cognitive beliefs that a specific outcome is more available following the presentation of a specific cue^15^.

Experiment 2 implemented an analogous instruction manipulation. After training and prior to the transfer phase, one group of participants was informed that the monsters had adapted to the learned defenses and that the *opposite* action should now be used for protection. In addition, we modified the transfer phase procedure to require a single response decision. During this phase, a CS was presented until the participant’s fight-or-flight decision (keypress) was registered. The dependent measure was the proportion of fight or flight decisions as a function of the presented cue. Consistent with the research of Seabrooke and colleagues, we hypothesized that this instruction should reverse the outcome-selective PIT effect observed under standard instructions^23,27^.

It is important to note that Experiment 2 originally included two additional conditions involving a cognitive load manipulation, modeled after the number order memory task used by Seabrooke and colleagues^27^. However, due to the online administration of this task, we were unable to confirm whether the task effectively induced cognitive load. As a result, we report here only the two conditions that did not include the memory task. Readers interested in the full set of analyses, including all experimental conditions, are referred to the Supplementary Information accompanying this article.

Experiment 3 examined the use of trained versus instructed cue-outcome associations using a threat induction procedure. Specifically, a high threat level was induced by informing participants that incoming monsters would be particularly fierce during certain periods of the transfer phase. Threat levels (high vs. low) associated with the presented monsters were manipulated within sessions, while instructions for the transfer test (standard vs. reversal) were varied between participants. Our dependent measure was the vigor of the fight or flight response (i.e., the response frequency), as in Experiment 1.

Based on the research of Seabrooke and colleagues^27^, we hypothesized that the PIT effect in the reversal instruction condition may be particularly attenuated in trials featuring highly threatening monsters, as stress may impair the complex rule-based processes required to override previously learned contingencies. Additionally, we anticipated that the outcome-selective PIT effect might also be reduced under standard instructions, consistent with prior findings demonstrating a negative relationship between specific PIT effects and emotional impulsivity at the trait level^28,29^. Accordingly, several predictions can be derived for this experiment.

## Results

### Experiment 1

**Fig. 1:**
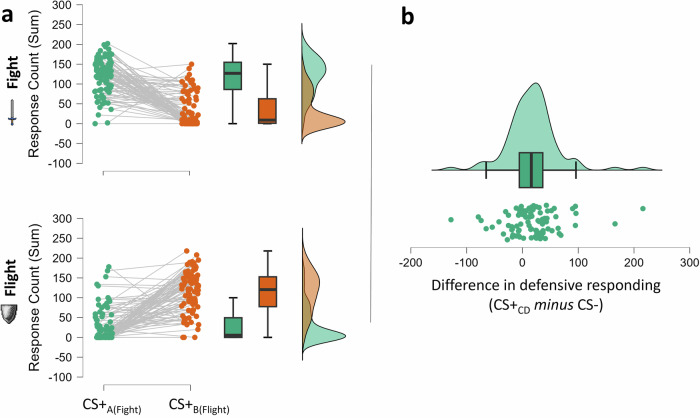
Specific and general PIT effects. **a** Counts of fight decisions (upper panel) and flight decisions (lower panel) as a function of the CS + . **b** Difference plot of aggregated counts of fight and flight responses. Higher scores (difference in response counts) indicate more defensive reactions during presentations of the CS + _CD_ relative to the CS-.

We hypothesized that presentations of the CS + _A(fight)_ would increase the number of fight responses, whereas presentations of the CS + _B(flight)_ would elevate flight responses, reflecting an outcome-specific PIT effect. To test this hypothesis, we conducted a 2$x$2 repeated measures ANOVA on cumulative response counts (sum score), with CS + _AB_ (fight vs. flight) and Response type (fight vs. flight) as within-subjects factors. The main effects of *CS* + _*AB*_*, F*(1, 85) = 1.20, *p* = 0.277, η_p_^2^ = 0.014, and of *Response Type, F*(1, 85) = 1.16, *p* = 0.285, η_p_^2^ = 0.013, were not significant. However, the hypothesized interaction effect was significant, *F*(1, 85) = 126.19, *p* < 0.001, η_p_^2^ = 0.598, demonstrating an outcome-specific PIT effect. As illustrated in Fig. 1, participants selected fight (*M* = 119.7, *SE* = 4.95) significantly more often than flight (*M* = 33.1, *SE* = 4.48) when exposed to CS + _A(fight)_. Conversely, they opted more frequently for flight (*M* = 113.3, *SE* = 5.61) than fight (*M* = 29.0, *SE* = 4.60) when exposed to CS + _B(flight)_.

In addition to the outcome-specific PIT effect, we hypothesized that participants would exhibit more vigorous defensive responses (i.e., cumulative counts of fight *and* flight responses) during presentations of CS + _CD_ relative to the CS-. A paired-samples *t*-test comparing response counts between these conditions revealed a significant difference, *t*(85) = 3.69, *p* < .001, *d*_z_ = 0.398, 95% CI [0.178, 0.617]. As hypothesized, participants engaged in defensive action more frequently when exposed to the CS + _CD_ (*M* = 288, *SE* = 7.89) compared to the CS- (*M* = 270, *SE* = 7.80), indicating a general PIT effect (see Panel b in Fig. 1).

### Interim discussion

Study 1 was designed to demonstrate both, outcome-specific and outcome-general transfer effects. Both effects were observed. The outcome-general PIT effect shows a general increase in defensive behaviors (fight-and-flight) following presentations of the novel-monster CS+ relative to the neutral CS-. This effect is in line with the extensive literature on the motivational excitation of defensive action by threat-conditioned cues^31,32^.

Even more interesting for the present research is the outcome-specific PIT effect. In the transfer phase, participants aligned their response choices to the contingencies between environments, appearances of specific monsters, and defensive actions against these monsters, selecting responses that were tailored to the specific threat signaled by the environments. The common element in both Pavlovian and instrumental associations was the specific monster that was paired as the threatening outcome with a particular environment in the Pavlovian phase and with a particular defensive action in the instrumental phase. The excitatory transfer from the CS to a specific defensive response can be explained by intersecting associations established during Pavlovian training—linking the CS (environment) to a specific US (monster)—and associations formed during instrumental training, where fight or flight from a specific monster was reinforced by the omission of an attack. Alternatively, this transfer might be attributed to a cognitive belief that a particular defensive action is a more effective response against attacks from the monster signaled by the environment.

### Experiment 2

#### Specific PIT

We analyzed the proportions of fight decisions (relative to flight) using a 2 (Instruction) $x$ 2 (CS + _AB_) mixed ANOVA. This analysis revealed a significant two-way interaction, *F*(1, 74) = 17.49, *p* < 0.001, η_p_^2^ = 0.191. The main effects of *CS* + _*AB*_, *F*(1, 74) = 1.47, *p* = 0.229, η_p_^2^ = .020, and *Instruction, F*(1, 74) = 2.73, *p* = 0.102, η_p_^2^ = 0.036, were not significant.

**Fig. 2:**
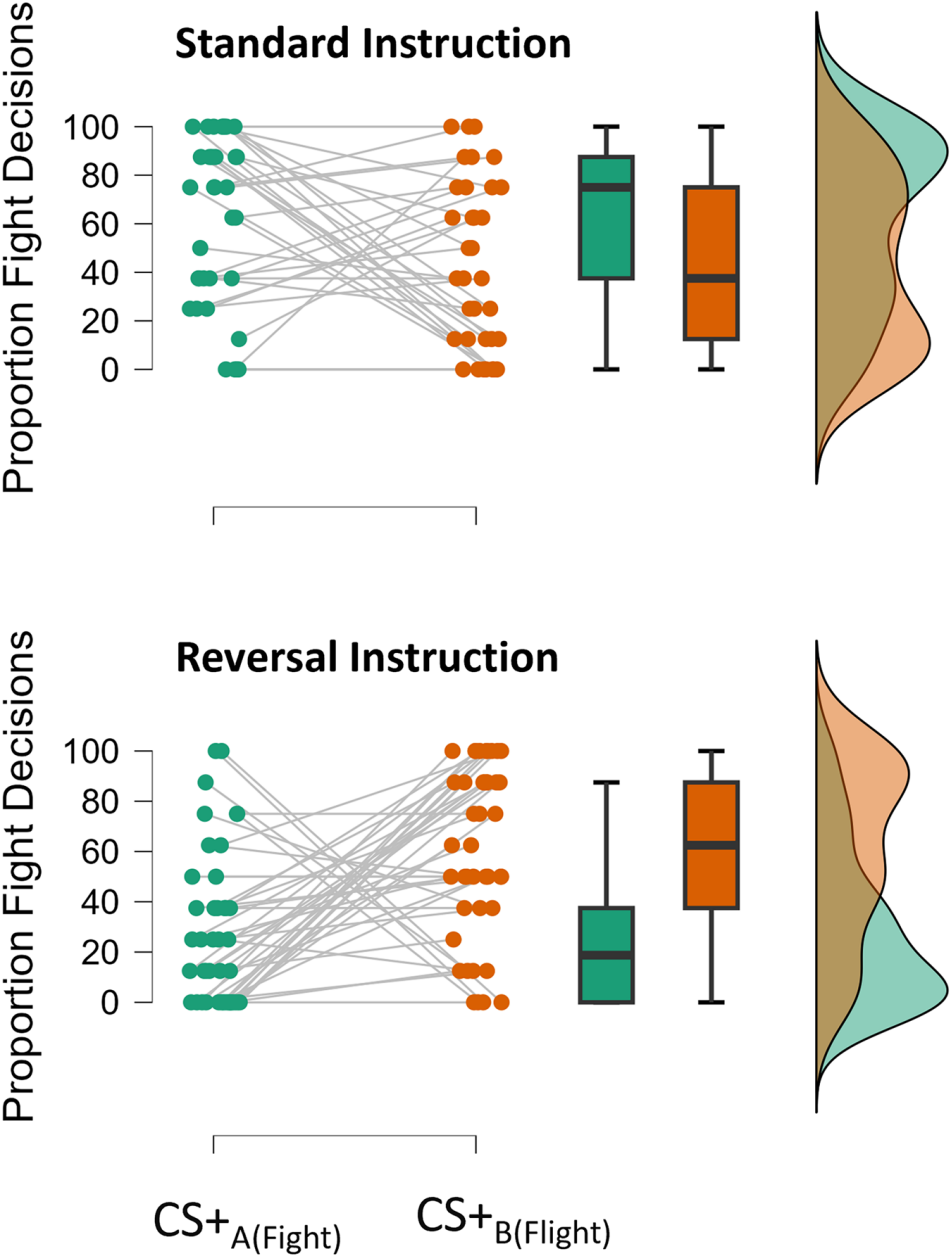
Specific PIT Effect As a Function of Post-Training Instruction in Study 2. Proportion of fight relative to flight decisions (in percent) as a function of the CS+ associated with fight or flight and the post-training instruction.

As depicted in Fig. 2, participants who received standard instructions exhibited an outcome-specific PIT effect, choosing to fight more frequently when exposed to CS + _A(fight)_ (*M* = 61%, *SE* = 5.6) compared to CS + _B(flight)_ (*M* = 43%, *SE* = 5.9). In contrast, participants who received reversal instructions displayed the opposite pattern: they chose to fight less frequently when exposed to CS + _A(fight)_ (*M* = 28%, *SE* = 5.0) than when exposed to CS + _B(flight)_ (*M* = 60%, *SE* = 5.3).

Exploratory analyses of action choices in response to the CS + _CD_ showed a relative preference for flight, as indicated by a lower proportion of fight responses (*M* = 48%, *SE* = 2.7) compared to the CS- baseline (*M* = 59%, *SE* = 3.1). A mixed ANOVA confirmed this difference, *F*(1, 74) = 5.05, *p* = 0.027, η_p_^2^ = 0.064. This action preference was not significantly influenced by the instruction manipulation (*F* < 1).

### Effectiveness ratings

As shown in Table 2, participants who received reversal instructions understood the manipulation and rated the opposite defensive action as more effective. However, a 2 (Instruction) $x$ 2 (CS + _AB_) mixed ANOVA revealed that the interaction effect was not statistically significant, *F*(1, 74) = 2.32, *p* = 0.102, η_p_^2^ = .036. The main effects were also not significant (*F*s < 1). Expectancy ratings for the *CS* + _*CD*_ and for the *CS-* were close to the midpoint of the rating scale, indicating no clear preference for either the fight or flight response.

**Table 2:** Means and standard deviations of effectiveness ratings in each instruction condition

	Standard Instruction	Reversal Instruction
CS + _A(Fight)_	2.76 (1.79)	3.41 (1.69)
CS + _B(Flight)_	3.03 (1.68)	2.63 (1.70)
CS + _CD_	3.32 (1.22)	3.21 (0.94)
CS-	2.85 (1.19)	2.95 (1.09)

Effectiveness ratings ranged on a bipolar scale from 1 (“fight”) to 3 (“neither”) to 5 (“flight”). There was one missing value in the reversal-instruction condition (*n* = 41).

### Interim discussion

Study 2 produced two main findings: First, a specific fight-or-flight PIT effect was found in the conditions with standard instruction. Participants opted more frequently for fight than for flight when the cue was associated with attackable monsters, and vice versa when the cue was associated with defensive retreat from monsters. This finding replicates the specific PIT effect observed in our first study using another dependent measure (fight-or-flight decision).

Second, the specific PIT effect was reversed by the reversal instructions to select the response opposite to the learned one. This finding conceptually replicates the results of Seabrooke and colleagues (2016) using an aversive PIT task. Moreover, expectancy ratings of flight or fight based on each cue were in line with the reversal instruction, suggesting the formation of new cognitive beliefs.

### Experiment 3

#### Specific PIT

Response frequencies (sum scores) were analyzed using a 2 (Instruction) $x$ 2 (Threat Level) $x$ 2 (CS + _AB_) $x$ 2 (Response) mixed ANOVA. *Instruction* was a between-subjects factor, while the remaining factors varied within subjects.

The main effect of *Threat Level* was significant, *F*(1, 81) = 7.21, *p* = 0.009, η_p_^2^ = 0.082. Participants exerted more effort for defense in trials featuring highly threatening monsters (*M* = 54.5, *SE* = 1.35) compared to trials with regular monsters (*M* = 53.3, *SE* = 1.31).

A significant two-way interaction effect between *Response* and *Threat Level* was observed, *F*(1, 81) = 7.12, *p* = 0.009, η_p_^2^ = 0.081, as well as a significant three-way interaction among *CS* + _*AB*_, *Response*, and *Threat Level*, *F*(1, 81) = 7.70, *p* = 0.007, η_p_^2^ = 0.087.

In trials with regular monsters, participant preferred fight (*M* = 61.7, *SE* = 4.6) over flight (*M* = 45.2, *SE* = 4.3) when exposed to CS + _A(fight)_ and flight (*M* = 60.4, *SE* = 3.9) over fight (*M* = 45.9, *SE* = 3.7) when exposed to CS + _B(flight)_.

In trials with highly threatening monsters, participants showed a stronger preference for flight (*M* = 64.2, *SE* = 3.6) over fight (*M* = 44.2, *SE* = 3.4) not only when exposed to CS + _B(flight)_ but also when exposed to the CS + _A(fight)_, where they still favored flight (*M* = 59.7, *SE* = 4.3) over fight (*M* = 49.7, *SE* = 3.6).

The hypothesized three-way interaction between *CS* + _*AB*_, *Response*, and *Instruction*, *F*(1, 81) = 101.80, *p* < 0.001, η_p_^2^ = 0.557, was significant, indicating a reversal of the PIT effect in the condition with reversal instructions. However, the hypothesized four-way interaction effect between *CS* + _*AB*_*, Response, Threat Level*, and *Instruction* was not significant, *F*(1, 81) = 0.17, *p* = 0.686, η_p_^2^ = 0.002. No other effect reached significance (*F*s ≤ 3.87, *p*s ≥ 0.053).

**Fig. 3:**
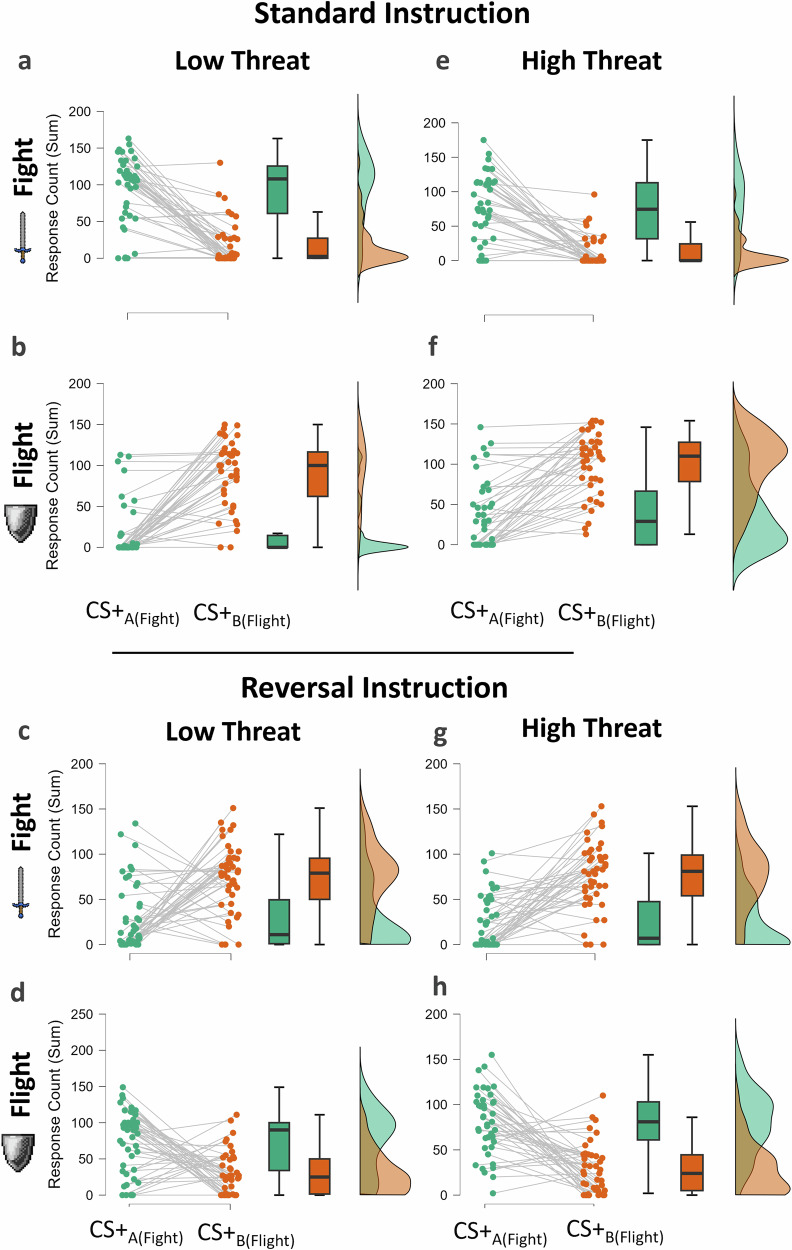
Specific PIT effect as a function of low and high threat levels in each instruction condition. Counts of fight responses (**a**, **c**, **e**, **g**) and flight responses (**b**, **d**, **f**, **h**) as a function of the CS+ in conditions with low threat (**a**–**d**) or high threat (**e**–**h**) and with standard instructions (**a**, **b**, **e**, **f**) or reversal instructions (**c**, **d**, **g**, **h**) for the transfer test phase.

Figure 3 illustrates the response pattern for each condition. Planned follow-up analyses, conducted separately for each instruction group using 2 (Threat Level) $x$ 2 (CS + _AB_) $x$ 2 (Response) ANOVAs, confirmed the hypothesized influence of task instruction on the direction of the outcome-selective PIT effects. When participants received standard instructions, they chose fight (*M* = 84.6, *SE* = 6.92) more frequently than flight (*M* = 28.3, *SE* = 5.31) when exposed to CS + _A(fight)_. Conversely, flight (*M* = 95.3, *SE* = 5.83) was preferred over fight (*M* = 15.4, *SE* = 3.44) when exposed to CS + _B(flight)_, *F*(1, 39) = 73.89, *p* < 0.001, η_p_^2^ = 0.655. This outcome-specific PIT effect was significantly larger when the threat level was low compared to high, *F*(1, 39) = 6.57, *p* = 0.014, η_p_^2^ = 0.144 (see the panels a, b, e, f in Fig. 3).

When the task instruction indicated a reversal of the fight-flight associations, the specific PIT effect was also reversed. In this condition, flight (*M* = 76.7, *SE* = 5.46) was preferred over fight (*M* = 28.8, *SE* = 4.56) when CS + _A(fight)_ was presented, whereas fight (*M* = 74.8, *SE* = 5.38) was chosen over flight (*M* = 29.5, *SE* = 3.94) when CS + _B(flight)_ was shown, *F*(1, 42) = 33.33, *p* < 0.001, η_p_^2^ = 0.442. The magnitude of this transfer effect did not differ between low- and high-threat conditions, *F*(1, 42) = 2.35, *p* = 0.133, η_p_^2^ = 0.053 (see the panels c, d, g, h in Fig. 3).

### Manipulation checks

Consistent with the intended manipulation of threat levels, the rate of defensive responding during presentations of CS + _CD_ was higher in trials with highly threatening monsters (*M* = 107, *SD* = 23.7) compared to trials with regular monsters (*M* = 104, *SD* = 23.8), *t*(82) = 2.69, *p* = 0.009, *d*_*z*_ = 0.29, 95% CI [0.07, 0.51]. Additionally, participants (*n* = 82, due to one missing value) reported significantly higher stress levels when the maleficent wizard appeared (*M* = 2.91, *SD* = 1.35) compared to when the benevolent wizard was shown (*M* = 1.45, *SD* = 0.69), *t*(81) = 9.54, *p* < 0.001, *d*_*z*_ = 1.05, 95% CI [0.78, 1.32].

Effectiveness ratings (*n* = 82, due to one missing value) were analyzed with a 2 (Instruction) $x$ 2 (CS + _AB_) mixed ANOVA. The main effects were not significant (*F*s < 1), but the interaction effect was significant, *F*(1, 80) = 23.29, *p* < 0.001, η_p_^2^ = .226. As shown in Table 3, participants in the reversal instruction group judged the alternative defensive action—opposite to the one rated as most effective by the standard instruction group—as more effective.

**Table 3:** Means and standard deviations of effectiveness ratings in each instruction condition

CS type	Standard Instruction	Reversal Instruction
CS + _A(Fight)_	2.33 (1.72)	3.79 (1.61)
CS + _B(Flight)_	4.08 (1.36)	2.63 (1.76)
CS + _CD_	3.58 (1.14)	3.16 (1.11)
CS-	3.06 (0.78)	2.83 (0.71)

Effectiveness ratings ranged on a bipolar scale from 1 (“fight”) to 3 (“neither”) to 5 (“flight”).

### Interim discussion

Our third study investigated the effects of reversal instructions and threat levels on outcome-specific transfer. In line with our previous studies, the instruction for the transfer phase had a large effect, demonstrating a reversal of the transfer effect with reversed cue-outcome relations.

The effect of our threat manipulation was more complex. When the threat level was high, participants generally opted more for flight than fight, and only less so when a fight cue was presented. In trials with a lower threat level, they selected the defensive action associated with the same outcome as the cue. Therefore, it appeared that the magnitude of transfer was reduced by high versus comparably low threat in the standard instruction conditions, whereas the threat level had no influence in the reversal instruction condition. However, the hypothesized four-way interaction effect was not significant. Therefore, it is plausible that high threat shifted the action priority to flight by a process independent of established cue-outcome beliefs.

## Discussion

In environments where danger is prevalent, the ability of organisms to detect signals indicating potential threats and to select appropriate actions in response to these threats is crucial for their survival. In the present research, the learning of threat cues was studied using Pavlovian conditioning, and the acquisition of defensive action knowledge was studied using instrumental conditioning. The motivating effect of Pavlovian threat cues on the use of action knowledge to either attack or flee from a source of threat was investigated using a transfer phase in which participants made fight-or-flight decisions in the presence of threatening cues signaling a monster that is attackable or from which one should better retreat.

Results of our first experiment showed that Pavlovian cues signaling a specific threat had a strong influence on action choice: participants preferred to fight when the threat cue signaled an attackable monster and to flee when the threat cue signaled a monster that is better avoided. This specific PIT effect shows that participants integrated Pavlovian knowledge about a specific source of threat with instrumental knowledge about coping with this specific threat for selecting an adaptive response.

Because conditioning phases were separate in our PIT paradigm, this transfer effect cannot be explained by a direct stimulus-response link but, rather, by the outcome shared by both types of associations. For the Pavlovian association, the outcome was the appearance of a dangerous monster, and for the instrumental association, attacking or fleeing from that monster. Hence, the common element was the association of a specific monster with a fight or flight response. By activating this knowledge, the cue signaling a danger (monster) primed a defensive action adaptive to this threat. A real-life example for this process would be encountering a feared person who was better shunned or confronted in previous social interactions. The expectation to meet this person at a specific place (the Pavlovian cue) would prime a confrontative, or avoidant interaction style depending on previous experiences with this person (the instrumental response), even if the person is encountered at this place for the first time (the transfer phase).

Experiments 2 and 3 investigated the role of propositional beliefs by reversing the cue-outcome relationship after training but before the transfer phase. Specifically, a cue previously associated with an attackable monster was redefined as signaling a threat best avoided, whereas a cue linked to an avoided monster now indicated a threat best confronted. These reversal instructions inverted the specific PIT effect observed under standard instructions, providing evidence that participants’ action choices were guided by cognitive beliefs about the most effective response to signaled threats. This conclusion is further supported by corresponding changes in participants’ ratings of the expected efficiency of defensive actions.

Our third experiment examined the influence of high and low threat levels on the magnitude of transfer. In certain trials, participants were warned of particularly severe monster attacks. The general increase in response rate and subjective self-reports show that participants were indeed more stressed in these trials. Crucially, the specific transfer effect was significantly weaker in high-threat trials, particularly under standard instructions, indicating that participants were less responsive to the specific threats signaled by the cues in these trials. This finding fits with other research demonstrating negative correlations between emotional impulsivity traits (chronic stress, negative urgency) and outcome-selective PIT effects^28,29^. In high-threat trials, an overall increase in defensive responses may reached a ceiling that impeded the detection of an additional outcome-selective response elevations^33^. With uncertainty about the success of a specific defensive action due to the extinction procedure, acting more vigorously and ignoring the predictive information from the cue could have been an adaptive response strategy that increased the likelihood of overall defense. Moreover, when anticipating particularly severe monster attacks, participants generally favored flight over fight. This shift toward flight may have stemmed from the belief that attacking extremely dangerous monsters carried a greater risk, potentially overriding prior conditioning-based associations. Further research is needed to determine the underlying mechanisms of this response shift and the reduced transfer effect in high-threat trials.

Overall, the present research findings reinforce the importance of cognitive beliefs in understanding the impact of Pavlovian cues on instrumental responding. Participants preferred the defensive action they learned as the most effective response to an anticipated threat and readily adapted their action choices when contingencies changed. Adaptive cognitions about the availability of outcomes, their value, and the efficacy of actions in attaining these outcomes are supported by numerous PIT studies with humans^34–37^ and they play a major role in goal-directed accounts of PIT^15,16,38^. These cognitions may be particularly important in environments with multiple and varying threats, where selecting the wrong action could be fatal.

The present research has several limitations. Threats were digital monsters and defensive actions were carried out in a digital game context without real-life implications for the individual’s health or well-being. We used this artificial setup due to ethical concerns, as did other studies before us^10,11^. However, it is possible that its artificiality has underrated emotional factors while overrating cognitive processes. To examine the generality and ecological validity of the results, future studies could aim to use less artificial stimuli, such as different types of pain.

Outcomes were threatening in both the instrumental and transfer phases, where defensive action against monster attacks was necessary to avoid being hit. In contrast, during the Pavlovian training phase, participants merely observed the monsters’ appearances without anticipating an attack. This procedural aspect may have led to the extinction of the attack expectancy. To reinstate this expectancy for the transfer test phase, a verbal instruction was used (“Defend yourself against attacks from invisible monsters”). It remains unclear whether the Pavlovian extinction of the monster-hit relationship influenced the results. In previous studies, Pavlovian extinction training (i.e., pairing the CS with no outcome) often failed to abolish outcome-selective PIT effects^35,39^. Further research is needed to determine whether this finding applies to the current paradigm.

Results are also limited by the technical use of keypresses for defense. A substantial body of evidence suggests that not all defensive behaviors are equivalent^1,40,41^. Some behaviors are learned more quickly in threatening contexts and/or are more difficult to control with cognitive beliefs^42^. Future research could explore the role of embodiment by incorporating whole-body movements, such as instructing participants to move forward for attack and backward for defense or vice versa^43,44^.

The learning conditions in the present study were also relatively simple, containing only small sets of stimuli and responses paired with outcomes using a fixed-ratio reinforcement schedule. Participants were trained until explicit knowledge of the cue-outcome and action-outcome relationships was perfect. It is hence not clear whether the present findings generalize to real-life conditions that are less ideal and more complex.

To conclude, the present studies demonstrate that people integrate information from predictive cues when making decisions to fight or flee, forming cognitive beliefs about the most effective response to a given threat. While these beliefs, established through conditioning procedures, can be readily updated when contingencies change, their application to behavior appears to be impeded in stressful situations.

## Methods

### Experiment 1

#### Transparency and openness

The research hypotheses, study procedures, data exclusion rules, and a target sample size of *N* = 90 participants were preregistered at 10.17605/OSF.IO/7XYN6. In the preregistration, the minimum sample size was calculated as N = 45 based on the specified parameters; however, this calculation was incorrect. In this article, the correct sample size is reported as N = 84. Importantly, this calculation error did not impact our preregistered sampling plan, which consistently aimed for N = 90 participants.

The experiments described in this article were performed with human subjects in accordance with the ethical standards as laid down in the 1964 Declaration of Helsinki and its later amendments. Informed consent was obtained from all participants. The study procedures were approved by the Institutional Review Board of the University of Würzburg (JMU), Institute for Psychology (reference no. GZEK 2015-08, GZ 2020-740).

### Sample

In the absence of an informed effect size estimate, we determined the sample size with a minimal effect size of interest in the range of *d*_z_ = 0.40 (for a justification of this choice, see ref. ^45^). According to the a-priori power analysis conducted using G*Power 3^46^, a minimum sample size of *N* = 84 participants is necessary to achieve high statistical power (1-β = 0.95) for the detection of an effect with *d*_z_ ≥ 0.40 in a two-tailed paired sample t-test with the alpha-level set to *p* = 0.05. Note that the squared t value of the paired t-test is equivalent to the *F*-value of a 2 × 2 repeated measures analysis of variance, resulting in identical *p*-values for both statistical tests. We preregistered to oversample to *N* = 90 in anticipation of a potential data loss (see our exclusion criteria below).

A total of *N* = 91 participants were recruited from Prolific (https://www.prolific.com/), for a monetary payment of ₤3.50. Participation required a physical computer keyboard. Median time for completing the study was about 30 minutes. In line with our preregistered exclusion criteria, three participants were excluded due to exclusive presses of a single key during the transfer phase ( ≥ 90% of the total number of keypresses), and an additional two participants due to no keypressing in a in a significant number of transfer test trials ( ≥ 33% of the trials). The final sample consisted of *N* = 86 participants (36 female, 50 male; age: *M* = 25 years, *SD* = 6.16, range: 18–55).

### Material

**Fig. 4:**
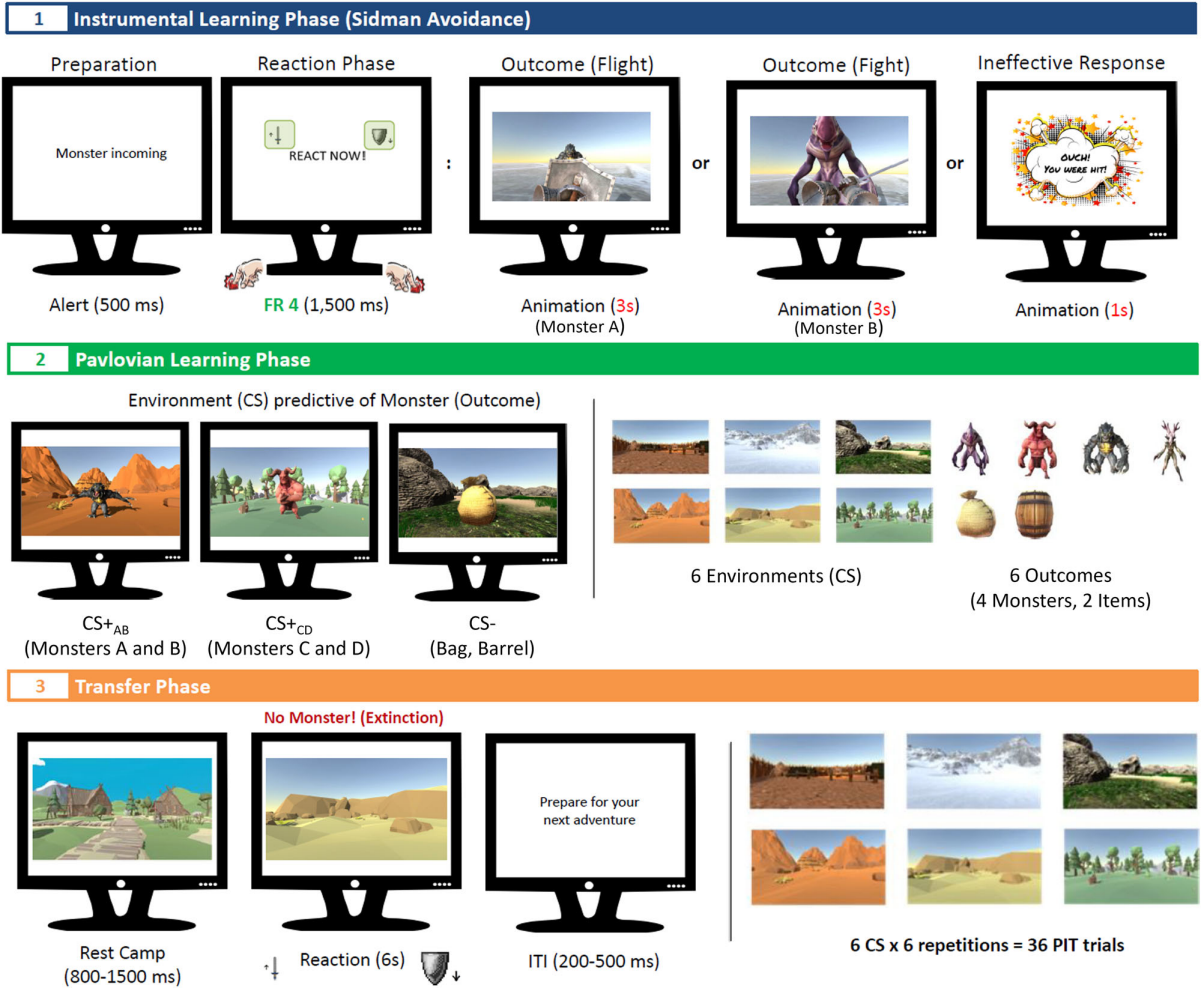
Experimental phases in study 1. (1) The Sidman avoidance training proceeded in two training blocks. In one block, attacking was effective (and fleeing ineffective), and in the other block, fleeing was effective (and attacking ineffective). Only one specific monster appeared in a block. The order of the training blocks was counterbalanced. (2) Environments signaling the appearance of a specific monster or item in the Pavlovian learning phase. (3) In the transfer phase, each environment was shown without the appearance of monsters and items (extinction). The video animations shown in the experiments can be viewed at https://osf.io/v3rkz.

The videos presented as action outcomes during the instrumental training phase were developed in-house using the Unity Development Platform. Each video (MPEG-4854×480 pixels) was crafted from an ego perspective, depicting either an attack animation with a sword swing while approaching the target or a defensive animation involving the raising of a shield while walking away from the target (refer to Fig. 4 for examples). The target in each video clip was an animated monster selected from a set of four distinct monsters (alien, demon, forest creature, gorosaur). Both attack and defensive retreat animations were created for each monster, resulting in a total of eight videos (four attack, four retreat). At the end of each animation, the monster disappeared, reinforcing the impression of either a successful attack or a successful retreat. Each video lasted three seconds.

For the Pavlovian training phase, a total of 48 video clips were created. Each video (MPEG-4, 854 × 480 pixels) showed a distinctive environment for about two seconds, selected from a set of six environments (see Fig. 4). Then, a monster or a neutral object (a fabric bag, a wooden barrel) appeared in the environment for an additional three seconds. Each monster exhibited a distinctive threatening gesture upon its appearance. The duration of each video was five seconds.

For the transfer phase, six pictures, one for each environment, were created. These pictures had a resolution of 1920 × 1080 pixels. An additional picture depicting a campsite was generated for presentation at the start of each trial.

The video clips can be viewed at https://osf.io/v3rkz/.

### Procedure

The experiment consisted of three phases: (1) the instrumental training phase; (2) the Pavlovian training phase, (3) and the transfer phase. Table 1 summarizes the experimental contingencies in each phase.

#### Phase 1

*Instrumental Training*. This phase comprised two training blocks, one featuring attack as the effective defense action (attack animation against Monster A) and the other with defensive retreat as the effective action (retreat animation against Monster B). The two remaining monsters, C and D, were not present in this phase (see Table 1). Each training block concluded after a fixed time of 180 s. The order of the blocks was counterbalanced. Instrumental training was modeled after a Sidman avoidance schedule, using a free-operant procedure adapted from Lewis and colleagues^10^. Instructions for this phase were:

“Welcome to the enchanted forest! As you stroll through the forest, you will encounter different types of monsters which will attack you! You goal is to survive lethal monster attacks! To survive, you can choose between attacking the monster or fleeing from the monster.”

Figure 4 shows the sequence of events in each trial. The participant was initially warned of an incoming monster (text message: “Monster incoming” for 500 ms), followed by a reaction prompt (text message: “React now!”). Participants could select between attacking the monster by pressing the upward arrow key or fleeing from the monster by pressing the downward arrow key. The key mapping was fixed to establish a spatial compatibility between the arrow keys and the forward (attack) or backward (retreat) motion implied by the actions^47^. The response window was set to 1500 ms. If the response key assigned to the correct defensive action was pressed at least four times during the response window (fixed ratio schedule, FR 4), the video displaying the corresponding defense animation was shown (duration: 3 s). In the event of another response (i.e., no or insufficient keypressing or presses of the ineffective key), a damage display (“Ouch! You were hit!”) appeared for 1 second, directly followed by the next trial. Consistent with a Sidman avoidance schedule, the selection of the effective defensive action, relative to the ineffective action, hence delayed incoming of the next monster by two seconds.

Task instructions were to find out which behavior is the most effective defense against a specific monster. Additionally, participants were explicitly informed about the avoidance schedule:

“If you chose the wrong action the monster will be unaffected by your action and directly attack again. If you chose the right action the monster will react and slow down! We will tell you when a monster is present. Once you see ‘react now’ it is your turn to defend yourself!”

Participants were thus explicitly warned about the presence of monsters. However, this warning signal did not provide any information about the type of monster or the specific defensive action required. Participants needed to infer this information by analyzing the contingencies between their actions and the resulting outcomes (for an overview, see Table 3). These outcomes were either a defense animation indicating successful defense or a message stating that the monster had hit the participant (see Fig. 4).

After each training block, contingency knowledge was tested. The contingency test consisted of a rating of the efficiency of each defensive action (fight vs. flight) on a numerical scale ranging from 1 (not at all) to 5 (extremely). The training block was repeated if the ineffective action for that block was rated as more efficient. This procedure was repeated until each contingency test was passed.

#### Phase 2: Pavlovian training

The task instructions for this phase emphasized that specific environments would attract specific monsters, and participants were to discern contingencies between environments and monster appearances. In each learning trial, a video was presented that showed a specific environment (see Fig. 4). After two seconds, a monster (or a neutral object) appeared in the environment, with the monster performing a threatening gesture (total duration of each video: 5 s). Participants were asked to simply observe the monsters appearing in each environment without executing a defensive action.

Participants viewed six videos, each depicting a different environment. Each environment was paired with either a monster (the two monsters, A and B, shown in the fight-flight videos of the instrumental phase and two novel monsters, C and D, that were not encountered before) or with a neutral object (a wooden barrel, a fabric bag). There were six blocks; in each training block, each video was presented in random order (6 videos x 6 blocks = 36 training trials).

After Pavlovian training, a contingency test was conducted to assess the participant’s knowledge of the environment-monster and environment-object relations. Participants were instructed to assign each environment to a specific monster or object via mouse clicks. Participants received immediate feedback about incorrect assignments, and a summary featuring all contingencies was presented at the end. After one or more incorrect assignments, the learning phase was repeated with half of the training trials (6 videos x 3 blocks = 18 trials), followed by a new contingency test. Upon passing this test, the participant proceeded to the next phase (the transfer phase). After two unsuccessful repetitions of the Pavlovian learning phase, however, the experiment was aborted, and this participant was dismissed.

#### Phase 3: Transfer phase

Task instructions for this phase stated that the adventurer (i.e., the participant) would now depart from a secured campsite to explore various areas, represented by pictures of specific environments. In each area, a threatening monster could spawn; however, the monster is now cloaked by an invisibility spell, meaning it cannot be seen in this phase. While exploring each area (i.e., during the presentation of an environment picture), they must defend themselves against the invisible monster with presses of the attack and retreat keys known from the previous phase. Instructions emphasized that the choice of the defensive action (fight vs. flight) is up to the participant, and that there would be no ‘correct’ action in this phase.

The sequence of events in this phase is shown in Fig. 4. Each trial started with the picture of a campsite (the adventurer’s resting place) displayed for a random duration between 800 and 1500 ms, followed by the environment picture for 6000 ms. Keypresses were registered during the presentation of the environment picture. Subsequently, the screen turned blank for a random intertrial interval between 200 and 500 ms, and the campsite reappeared, initiating the next trial. The transfer phase consisted of six blocks, each presenting each environment picture once in random order, resulting in a total of 36 trials.

After the transfer phase, participants completed the Buss-Perry Aggression Questionnaire for exploratory analyses^48^. Then, they were thanked, debriefed about the purpose of the study, and referred back to Prolific for payment.

### Design

The experiment had a 3$x$2 factorial design with *Cue Type* and *Response Type* as within-factors.

Cue type varied at three levels:

1. *CS* + _*AB*_*:* Environments (CSs) paired with Monsters A and B presented during the instrumental phase. To further differentiate, *CS* + _*A(fight)*_ refers to the CS+ paired with the monster associated with a fight response, and *CS* + _*B(flight)*_ to the CS+ paired with the monster associated with a flight response.
2. *CS* + _*CD*_*:* Environments (CSs) paired with the new Monsters C and D during the Pavlovian phase.
3. *CS-:* Environments (CSs) paired with the neutral items (bag, barrel) during the Pavlovian phase.

Response type referred to fight-or-flight choices. The dependent variable of interest was the frequency of keypresses during CS presentations in the transfer phase (i.e., the cumulative sum of the keypresses as a function of cue type).

The order of the instrumental training blocks (initial training of the fight-response vs fight response) was counterbalanced across participants. The assignment of the monsters/objects to the environments was random.

### Experiment 2

#### Transparency and openness

The research hypotheses, study procedures, data exclusion rules, and a target sample size of *N* = 150 participants were preregistered at 10.17605/OSF.IO/WYZQC. The preregistered experiment included two additional conditions with a number recall task for manipulating cognitive load. However, analyses of the performance in this task revealed that most participants performed exceptionally well on the task, raising questions about the validity of the cognitive load task that was administered online. After careful consideration, we chose not to include these conditions in the present report. The preregistered analyses, including all four experimental conditions, are available in the Supplementary Information accompanying this article.

### Sample

We hypothesized a significant three-way interaction effect between CS type, Cognitive Load and instruction groups. The a-priori power analysis conducted with G*Power 3 showed that a minimum sample size of *N* = 144 would have sufficient statistical power (1-β = 0.95) to detect a medium-sized within-between interaction effect (f ≥ 0.25) with zero correlation between the repeated measures. Anticipating potential dropouts, we planned to collect data from *N* = 150 participants.

A total of *N* = 152 participants were recruited from Prolific. Participants received a fixed payment of ₤4.00 and a bonus payment of ₤0.50 for sufficient performance in the number recall task ( ≥50% correct answers). Participation required a physical computer keyboard and audio equipment. Median time for completing the study was about 40 minutes. Following our preregistered exclusion criteria, nine participants were excluded due to the exploitation of a response strategy during the transfer phase (same action decision in ≥90% of the trials). No exclusions were necessary based on the performance in the number recall task. The final sample consisted of *N* = 143 participants (56 female, 86 male, 1 diverse/non-binary/other; age: *M* = 32.2 years, *SD* = 11.3, range: 18-69). From this sample, *n* = 34 participants were assigned to the standard condition without load, *n* = 33 to the standard condition with cognitive load, *n* = 42 to the reversal-instruction condition without load, and *n* = 34 to the reversal-instruction condition with cognitive load. Only the two conditions without cognitive load were retained for the analyses reported in this article.

### Material

Video clips and pictures were the same as in Study 1. Audio tracks featuring a female voice vocalizing numbers from 1 to 9 in American English were taken from the software package Inquisit™ (length ≈ one second per number).

### Procedure

The study procedure was modified in the following way:

#### Changes to Phase 1: Instrumental training

Each session started with two instrumental training blocks which were identical to those of Study 1 with one exception: Instead of four keypresses, only a single key press was necessary to generate the response-contingent flight or fight animation (FR1).

#### Changes to Phase 2: Pavlovian training

None.

#### Changes to Phase 3: Transfer phase

Instructions for the transfer phase varied depending on the assigned condition (see *Instruction Manipulation*). In addition, participants completed either a number recall task or a filler task (see *Cognitive Load Manipulation*).

In each trial, the campsite picture first appeared for about 8 s, followed by a blank screen for 500 ms. Then, an environment picture (CS) was shown until registration of the participant’s flight-or-fight decision (keypress). Subsequently, participants completed the number recall task (load condition) or a filler task prompting the press of a designated number key (no load condition). The trial ended with a blank screen shown for 200–500 ms. In total, participants completed eight transfer blocks with six trials each (one per CS) in random order, resulting in 48 trials.

#### Instruction manipulation

Participants assigned to the condition with standard instructions received the same instructions as in Experiment 1. In the conditions with reversal instructions, a cartoon wizard stated prior to the transfer phase that the monsters had adapted to defensive actions and recommended using the opposite action. During the transfer phase, participants were constantly reminded of the wizard’s recommendation by a written statement beneath the environment picture (“The learned action is ineffective! Try the other action!”).

#### Cognitive load manipulation

For the induction of cognitive load during the transfer phase, the number order memory task of Seabrooke and colleagues (2019) was used. Participants heard six different numbers randomly selected from 1 to 9 (without repetitions) with an interstimulus interval between 200 and 330 ms (depending on the length of the audio stimulus). Participants were tasked with memorizing the sequence of the six numbers as well as they could. After the participant’s action decision, a number prompt appeared that asked for a randomly selected number in the sequence except for the last one (‘Which number came after [number]?’). Participants entered the answer by pressing the corresponding digit key on the keyboard. Error feedback was presented if the entered number was incorrect (‘Incorrect. You chose the wrong number’). Otherwise, no message appeared.

In the conditions without cognitive load, the timing was identical, but participants heard no numbers and only saw the campsite image. Random number sequences were actually drawn as in the load condition, and audio files were played with zero volume to ensure identical timing. Instead of asking for a memorized number, participants were prompted to press a prespecified number key (e.g., “Press the key with the number 7”). Error feedback was identical to the load condition if the keypress was incorrect.

The number recall task was practiced with ten trials before the instrumental training (Phase 1). These practice trials were identical to the number recall task used for the transfer phase, but without CS presentations and fight-or-flight decisions. For task practice, instructions prompted a corresponding keypress (“Press the [up, down] arrow key”) in 50/50 ratio, mimicking a response decision. Participants assigned to the conditions without cognitive load did not practice the number task and started directly with the instrumental training.

A monetary incentive was provided for sufficient performance in the number recall task, with a bonus payment of £0.50 awarded for correct recall in more than 50% of the trials. To ensure equal payment across conditions, a bonus payment was also given to participants in the conditions without cognitive load if the proportion of correct keypresses surpassed 50%. Task instructions informed participants about the bonus payment without specifying a particular performance criterion (instead referring to “good performance”).

After the transfer phase, participants rated the effectiveness of fight or flight actions against monsters depending on the environment (Question: “When this environment was presented, which response do you think was more effective?”). Each CS was presented again and the participant rated the effectiveness of fight relative to flight in this environment on a bipolar scale ranging from 1 (“fight”) to 3 (“neither”) to 5 (“flight”). Subsequently, they completed the Buss-Perry Aggression Questionnaire and were dismissed.

### Experiment 3

#### Transparency and openness

The research hypotheses, study procedures, data exclusion rules, and a target sample size of *N* = 80 participants were preregistered at 10.17605/OSF.IO/C2MSX.

### Sample

The hypotheses predicted a four-way interaction effect between cue type, response type, reversal instructions, and threat level. To reduce the complexity of the power analysis, statistical power was planned with PIT effects computed for each threat level in each instruction condition (resulting in four PIT effects). For the corresponding 2 (threat) x 2 (instruction) mixed ANOVA, we hypothesized a significant within-between interaction effect that indicates different magnitudes of PIT effects depending on the threat level in each instruction condition. Using the effect size of the four-way interaction effect in Study 2 for an informed effect size estimate (η_p_^2^ = 0.081), a power analysis conducted with G*Power 3 revealed that a minimum sample size of *N* = 70 would be needed to detect a within-between interaction effect with η_p_^2^ = 0.081 (or f ≥ 0.296) with high statistical power (1-β = 0.95) in a corresponding 2 × 2 mixed ANOVA test with the correlation between the repeated measures set to 0.1 and the alpha-level set to 0.05. In the anticipation of potential dropouts, we planned to oversample to a minimum of *N* = 80 and to replace dropouts until the specified sample size was reached.

A total of *N* = 87 participants were recruited via Prolific. Participants received ₤5.00 for completing the experiment and a bonus payment for succeeding in the task (see the Threat Manipulation below). Participation required a physical computer keyboard and audio equipment. In line with our preregistered exclusion criteria, four participants were excluded due to exclusive presses of a single response key during the transfer phase ( ≥ 90% of the total number of keypresses). The final sample consisted of *N* = 83 participants (38 female, 45 male; age: *M* = 29.7 years, *SD* = 9.2, range: 18–66). From this sample, *n* = 40 participants were assigned to the standard condition and *n* = 43 to the reversal-instruction condition.

### Material, design, and procedures

The video clips and pictures from Study 1 were utilized. The experiment featured a mixed 2 × 2 design, with *Threat Level* (high vs. regular) as a within-subjects variable and *Instruction* (standard vs. reversal) as a between-subjects variable. Participants were randomly assigned to one of the instruction groups.

#### Changes to Phase 1: Instrumental training

The experiment commenced with two instrumental training blocks, mirroring those in Study 1, but with the following modifications: (1) Participants were required to press a response key eight times within a 1500 ms window to trigger a defensive action animation (FR8). (2) During the instrumental phase, the screen displayed three red heart symbols, signifying the health of the participant’s avatar. Failing to respond correctly in three trials resulted in the avatar’s health bar decreasing by half a heart. (3) Participants who did not pass the contingency test after retraining were dismissed.

#### Changes to Phase 2: Pavlovian training

The Pavlovian training was identical to that in Study 1.

#### Changes to Phase 3: Transfer phase

The transfer phase consisted of eight blocks, each featuring presentations of the six CS (totaling 48 trials). For half of the trials, a cue signaled that the monster would be particularly fierce (high threat). The order of the trials with fierce and regular monsters was randomized.

#### Threat manipulation

**Fig. 5:**
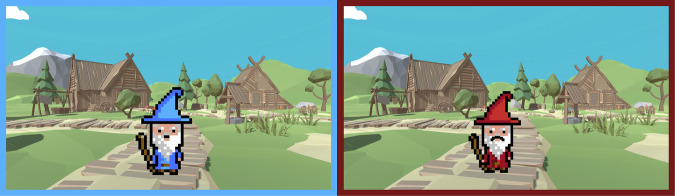
Wizards signaling the threat level of incoming monsters. The benevolent wizard is shown on the left and the maleficent wizard on the right.

Instructions for the transfer phase informed the participant that an evil wizard dressed in red would sporadically appear who would magically strengthen the incoming monsters. The campsite, shown initially, featured either the benevolent wizard (in blue) or the malevolent wizard (in red), indicating the monsters’ dangerousness in this trial (see Fig. 5). Each environment (CS) was paired four times with each wizard, resulting in 24 trials per threat condition.

To further elevate threat levels, participants were told that ineffective defensive actions against the monsters would diminish the avatar’s health (similar to the instrumental phase). Additionally, it was mentioned that the participant would receive a monetary bonus if they kept the avatar alive. However, it was also stated that feedback on monster hits or the avatar’s health status would be unavailable during this phase (in line with an extinction test). Instead, a text message (“Stay alive! React now!”), along with an image of a heart with a superimposed question mark and a golden star, was displayed at the top of the computer screen. This served as a constant reminder of the risks to the avatar’s health and the urgency for defensive actions- Regarding the bonus payment, no specific performance criterion was stated beyond the general objective of keeping the avatar alive. However, in practice, the bonus ₤1.00 was awarded to all participants, regardless of their actual task performance.

#### Instruction manipulation

The reversal instructions were the same as those utilized in Study 2.

#### Effectiveness and threat ratings

After the transfer phase, participants rated the effectiveness of fight or flight actions against monsters depending on the shown environment on five-point scales using the procedure of Study 2. Then, the pictures of the campsites featuring the benevolent and maleficent wizards were presented and participants were to indicate how stressed they were when this camp was presented (1 = “not at all”; 3 = “medium”; 5 = “extremely”). Subsequently, they completed the Buss-Perry Aggression Questionnaire and were dismissed.

## Supplementary information

Supplementary Information

## Data Availability

Preregistration documents, stimulus material (video clips) and raw data are available at https://osf.io/v3rkz/.

## Code availability

Data were analyzed with SPSS Statistics Version 27. Scripts used for the data analyses are available at https://osf.io/v3rkz/.
