# Supplementary material for: Pavlovian to instrumental transfer of control over fight or flight decisions
Source: NPJ Sci Learn. 2025 May 28;10:34. doi: 10.1038/s41539-025-00331-4 (PMC12119884; doi:10.1038/s41539-025-00331-4)
Supplement: Supplementary file 1 — Supplementary Information [file 41539_2025_331_MOESM1_ESM.pdf]

Supplementary Information

Supplementary to Experiment 2

The original experiment, preregistered at https://doi.org/10.17605/OSF.IO/WYZQC, included two additional conditions with a cognitive load manipulation. Specifically, participants were randomly assigned to one of four conditions:

1. A condition with standard instructions for the transfer phase and without cognitive load (standard no load condition)
2. A condition with standard instructions for the transfer phase and with cognitive load (standard load condition)
3. A condition with a reversal instruction for the transfer phase and without cognitive load (reversal no load condition)
4. A condition with a reversal instruction for the transfer phase and with cognitive load (reversal load condition)

Reversal instructions for the transfer phase stated that the monsters have adapted to the learned defense and that the opposite action should be used for defense. We used the same cognitive load manipulation (a number order memory task) as Seabrooke and colleagues [1]. With respect to this manipulation, we reasoned that cognitive load would primarily impair response decision and not the execution of the selected response. Therefore, we changed the transfer phase procedure to require a single response. During the transfer phase, a CS was shown until the participant's fight-or-flight decision (keypress) was registered. The dependent measure was the proportion of fight or flight decisions as a function of the presented cue. Although new-monster CS+ were still shown during the transfer phase, this procedural change precluded the analysis of a general PIT effect, because participants could not opt against selecting a defensive response in

the trials. In line with Seabrooke and colleagues [1], we hypothesized a specific fight-or-flight PIT effect for the conditions with standard instructions, which would be robust against the cognitive load manipulation. In contrast, we expected no PIT effect for the reversal load condition and a reversed PIT effect in the reversal no-load condition.

The method, including a description of materials and task procedures, is reported in the main article.

Supplementary Results

Specific PIT

We analyzed the proportions of fight decisions (relative to flight) using a 2 (Instruction) *x* 2 (Cognitive Load) *x* 2 ($CS+_{AB}$) mixed ANOVA. This analysis revealed a significant two-way interaction between $CS+_{AB}$ and *Instruction*, $F(1, 139) = 93.45$, $p < .001$, $\eta_p^2 = .402$, as well as a significant two-way interaction effect between $CS+_{AB}$ and *Cognitive Load*, $F(1, 139) = 6.93$, $p = .009$, $\eta_p^2 = .047$. The hypothesized three-way interaction effect between $CS+_{AB}$, *Instruction*, and *Cognitive Load* was significant, $F(1, 139) = 12.12$, $p < .001$, $\eta_p^2 = .080$.

As depicted in Supplementary Figure 1, participants receiving standard instructions and without cognitive load exhibited an outcome-specific PIT effect, opting more frequently for fighting when exposed to the $CS+_{A(fight)}$ ($M = 61\%$, $SE = 5.5$) compared to $CS+_{B(flight)}$ ($M = 43\%$, $SE = 5.2$). This effect was amplified under cognitive load ($CS+_{A(fight)}$: $M = 83\%$, $SE = 5.6$; $CS+_{B(flight)}$: $M = 15\%$, $SE = 5.3$).

When receiving reversal instructions, participants' action choices were opposite to the CS+ flight-fight association. Without cognitive load, they opted for fight less frequently when exposed to the $CS+_{A(fight)}$ ($M = 28\%$, $SE = 4.9$) compared to the $CS+_{B(flight)}$ ($M = 60\%$, $SE = 4.7$). This

reversed pattern persisted under cognitive load (CS+$_{A(fight)}$: M = 35%, SE = 5.5; CS+$_{B(flight)}$: M = 75%, SE = 5.2).

The omnibus analysis indicated no significant main effects (Fs ≤ 1.34, ps ≥ .249). However, the interaction between *Instruction* and *Cognitive Load* was significant, $F(1, 139) = 4.50$, $p = .036$, $\eta_p^2 = .031$.Under cognitive load, participants receiving the reversal instruction opted more frequently for fight than for flight, whereas those in the standard instruction condition exhibited a preference for flight over fight.

Supplementary Figure 1

Specific PIT Effect in Each Condition of Study 2

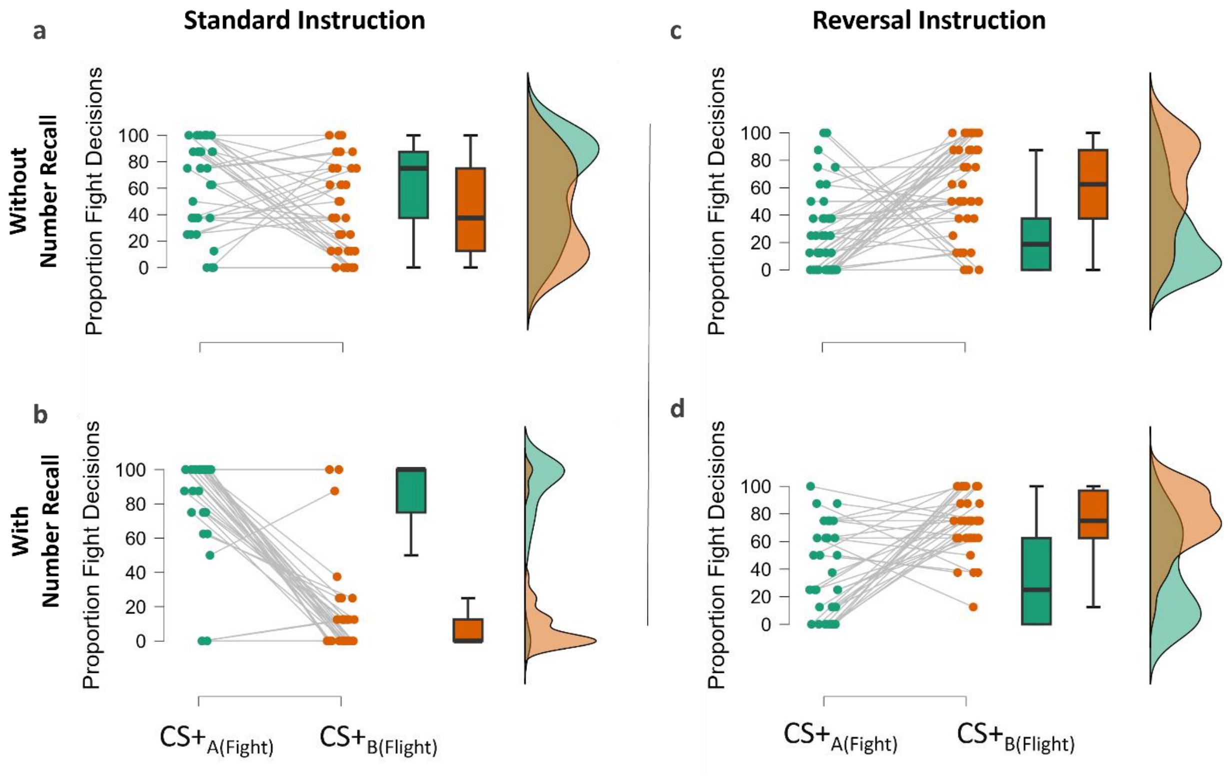

Note. Proportion of fight relative to flight decisions (in percent) as a function of the CS+ with post-training standard instructions (a, b) and reversal instructions (c, d) for transfer test conditions without the number recall task (a, c) and with the number recall task (b, d).

Exploratory analyses of action choices when exposed to the $CS+_{CD}$ revealed no preference for a specific defensive action ($M = 51$ %, $SD = 24.3$). Additionally, action choices to this cue were not significantly influenced by cognitive load or the instruction manipulation (Fs $\leq$ 2.90, ps $\geq$ .091).

Number recall task

Performance in the number recall task in the load conditions was very high and did not differ between groups with standard ($M = 88.5\%$, $SD = 13.1$) and reversal instructions ($M = 91.5\%$, $SD = 11.2$) according to a Welch's t-test, $t(63.2) = 1.23$, $p = .271$, $d_s = 0.272$. Across all conditions, the percentage of correct answers was $Md = 95.8\%$, $IQR = 12.5$, Min-Max: 54–100%. These results suggest that most participants performed exceptionally well on the task, raising questions about the validity of the cognitive load manipulation.

Effectiveness ratings

A 2 (Instruction) x 2 (Cognitive Load) x 2 ($CS+_{AB}$) mixed ANOVA revealed a significant two-way interaction between $CS+_{AB}$ and *Instruction*, $F(1, 139) = 43.56$, $p < .001$, $\eta_p^2 = .239$, as well as a significant three-way interaction, $F(1, 139) = 16.39$, $p < .001$, $\eta_p^2 = .105$. All other effects were not significant, Fs ≤ 3.80, ps $\geq .116$.

As shown in Supplementary Table 1, participants who received the reversal instructions understood the manipulation and rated the opposite defensive action as more effective under these conditions. These beliefs were more strongly expressed by participants in conditions with cognitive load. Expectancy ratings for the $CS+_{CD}$ ($M = 3.2$, $SD = 1.0$) and for the *CS-* ($M = 2.8$, $SD = 1.2$) favored neither the fight nor flight response.

Supplementary Table 1

Means and Standard Deviations of Effectiveness Ratings in Each Condition

	Standard Instruction		Reversal Instruction	
CS type	Without Load	With Load	Without Load	With Load
$CS+_{A(Fight)}$	2.76 (1.79)	1.76 (1.52)	3.41 (1.69)	4.12 (1.25)
$CS+_{B(Flight)}$	3.03 (1.68)	4.55 (1.06)	2.63 (1.70)	2.32 (1.47)
$CS+_{CD}$	3.32 (1.22)	3.14 (1.14)	3.21 (0.94)	3.0 (0.71)
CS-	2.85 (1.19)	2.35 (1.33)	2.95 (1.09)	3.0 (0.97)

Note: Effectiveness ratings ranged on a bipolar scale from 1 (“fight”) to 3 (“neither”) to 5 (“flight”). There was one missing value in the reversal-instruction condition without load (n = 41).

Supplementary Discussion

Study 2 produced three main findings: First, a specific fight-or-flight PIT effect was found in the conditions with standard instruction. Participants opted more frequently for fight than for flight when the cue was associated with attackable monsters, and vice versa when the cue was associated with defensive retreat from monsters. This finding replicates the specific PIT effect observed in our first study using another dependent measure (fight-or-flight decision).

Second, the specific PIT effect was reversed by the reversal instructions to select the response opposite to the learned one. This finding conceptually replicates the results of Seabrooke and colleagues[2] using an aversive PIT task. Moreover, expectancy ratings of flight or fight based on each cue were in line with the reversal instruction, suggesting the formation of new cognitive beliefs.

Third, the specific PIT effect obtained with standard instructions was larger in the condition with cognitive load compared to no load, whereas cognitive load did not affect the magnitude of transfer in the conditions with reversal instructions. However, this finding is questionable because, due to the online administration of the number recall task, we could not verify whether the task was correctly administrated in inducing cognitive load. For example, participants could simply have recorded the numbers during listening, which could account for the exceptionally high performance observed in this task. Consequently, we cannot determine whether the observed differences across conditions were attributable to cognitive load or alternative strategies employed by participants. Therefore, we refrain from interpreting the differences in the standard conditions as being causally influenced by cognitive load.

Supplementary References

1. Seabrooke, T., Wills, A. J., Hogarth, L. & Mitchell, C. J. Automaticity and cognitive control: Effects of cognitive load on cue-controlled reward choice. *Quarterly Journal of Experimental Psychology* **72**, 1507–1521 (2019).
2. Seabrooke, T., Hogarth, L. & Mitchell, C. J. The propositional basis of cue-controlled reward seeking. *The Quarterly Journal of Experimental Psychology* **69**, 2452–2470 (2016).
